# Brown Seaweed (*Padina australis*) Extract can Promote Performance, Innate Immune Responses, Digestive Enzyme Activities, Intestinal Gene Expression and Resistance against *Aeromonas hydrophila* in Common Carp (*Cyprinus carpio*)

**DOI:** 10.3390/ani12233389

**Published:** 2022-12-02

**Authors:** Najmeh Sheikhzadeh, Ehsan Ahmadifar, Mehdi Soltani, Hossein Tayefi-Nasrabadi, Shalaleh Mousavi, Mohammed A. E. Naiel

**Affiliations:** 1 Department of Food Hygiene and Aquatic Animals, Faculty of Veterinary Medicine, University of Tabriz, Tabriz 51666-14766, Iran; 2 Department of of Fisheries, Faculty of Natural Resources, University of Zabol, Zabol 98613-35856, Iran; 3 Department of Aquatic Animal Health, Faculty of Veterinary Medicine, University of Tehran, Tehran 14155-6453, Iran; 4 Centre for Sustainable Aquatic Ecosystems, Harry Butler Institute, Murdoch University, Murdoch, WA 6150, Australia; 5 Department of Basic Sciences, Faculty of Veterinary Medicine, University of Tabriz, Tabriz 51666-14766, Iran; 6 Department of Animal Production, Faculty of Agriculture, Zagazig University, Zagazig 44519, Egypt

**Keywords:** brown algae, common carp, disease challenge, immunity

## Abstract

**Simple Summary:**

Fish farming is threatened by various stressors involved in impairing fish health and productivity. Hence, environmentally friendly feed additives such as seaweeds or their extracts could be an effective alternative to antibiotic therapy. *Padina australis* is a brown seaweed spread on the coasts of Iran and characterized by its abundant supply of several bioactive molecules and acids. Thus, this study was performed to evaluate its functional roles in the rate of growth, immune responses, and disease tolerance of common carp towards *Aeromonas hydrophila* infection. It could be concluded that dietary *P. australis* at levels of 100 up to 400 mg/kg diet could promote the growth and general health status of common carp.

**Abstract:**

Eight-week oral administration of *Padina australis* ethyl acetate extract at 100, 200, and 400 mg/kg diets was assessed on the growth performance, tight junction proteins, intestinal immunity, and disease resistance to *Aeromonas hydrophila* in common carp (*Cyprinus carpio*). A total of 300 healthy common carp weighing around 14.8 ± 0.03 g were randomly assigned into four equal groups within 12 glass aquariums, each in three replicates (25 fish/tank), for the feeding trial experiment. The first group served as the control group and was fed an un-supplemented diet, whilst the other three groups were offered diets containing graded amounts of *Padina australis* ethyl acetate extract at 100, 200, and 400 mg/kg, respectively. The growth indices, including final weight, length, weight gain rate, specific growth rate, and feed conversion ratio, were meaningfully improved in fish fed with the algae at 200 and 400 mg/kg compared to the control fish (*p* < 0.05). Similarly, digestive enzyme activities and serum immune parameters were significantly higher in all treatments, especially 200 and 400 mg/kg fed groups, compared to the control (*p* < 0.05). In parallel, significant upregulation of genes related to integrity and the immune system was shown in the intestine of these treatment groups compared to control fish (*p* < 0.05). When fish were challenged with *A. hydrophila*, the cumulative survival percentages were 53.3% (*p* = 0.215), 70.0 % (*p* = 0.009), and 76.7% (*p* = 0.002) in fish fed 100, 200, and 400 mg/kg diets, respectively, compared to 36.7% survival in control fish (*p* = 0.134). These data show that the eight-week dietary administration of *P. australis* extract to common carp can enhance growth performance, digestive enzyme activity, immune response, and disease resistance to *A. hydrophila* infection.

## 1. Introduction

One of the most extensively cultured freshwater fish species is the common carp (*Cyprinus carpio* L.), with a high potential for intensive and super-intensive fish farming systems [1]. However, fish farming is threatened by various stressors impairing fish health and productivity [2,3]. Abiotic and biotic farming systems can mainly induce oxidative stress followed by immunosuppression, thereby bringing about a high vulnerability to infection by pathogenic invaders [4,5,6]. *A. hydrophila* infection is a predominant pathogenic bacterium in carp farms that causes motile *Aeromonas* septicemia with high morbidity and mortality [7]. In case of infection, chemotherapies are usually prescribed to relieve the impacts of *A. hydrophila* infection on common carp [8]. Although fish farmers are currently using some antibiotics for the treatment/control of this bacterial disease, a continuous supply of antibiotics can enhance bacterial resistance, environmentally hazardous factors, and food safety issues [9,10]. Hence, antibiotics may be substituted with ecologically safe feed additives such as probiotics/prebiotics, seaweeds, and herbal drugs [11,12,13].

Seaweeds and their extracts are recognized as natural active supplements in aquaculture [14,15,16]. Red, green, and brown seaweeds are applied in the human food, animal feed and pharmaceutical industries [17,18,19]. In aquaculture, seaweeds also are incorporated in the aqua feed for their influential role as growth promoters and for possessing antibacterial, antioxidative, and immunostimulant properties [20,21]. *P. australis* is a brown seaweed spread on the coasts of Iran and characterized by its abundant supply of flavonoids, terpenoids, alkaloids, steroids, tannins, polyphenols, fucoxanthin, saponins, and fatty acids [22,23]. Recently, Salosso, et al. [24] demonstrated that *P. australis* extract had high levels of phenols, tannin, flavonoid, and steroids, which may be responsible for suppressing *A. hydrophila* in vitro. Additionally, Akbary and Aminikhoei [25] reported that 1.0 g/kg of water-soluble polysaccharide extract of *P. australis* is beneficial to *L. vannamei* growth indices, antioxidant, and non-specific immunological responses. Furthermore, supplementing grey mullet diets with graded amounts of *P. astraulis* extract improved lipid and carbohydrate metabolism while also increasing digestive enzyme activity [26]. Despite the broad spectrum of beneficial components in this seaweed, the few studies that have been performed on aquatic organisms, including grey mullet (*Mugil cephalus*) [27,28], exhibit a promising effect on their growth and health status. However, minimal data are available on the efficacy of this algae, either in the form of powder or extract, on the immuno-physiological variables of fish. Because this algae is widely accessible in Iran′s coastal areas and is inexpensive to use as a fish feed supplement, this study was performed to evaluate its functional roles in the rate of growth, immune responses, and disease tolerance of common carp towards *A. hydrophila* infection.

## 2. Materials and Methods

### 2.1. P. australis Extract Preparation

*P. australis* was collected from the Bushehr coast (Persian Gulf, Bukht Ardashir, Iran) in December 2020. The algae were validated at the University of Khoramshahr herbarium and recognized as *P. australis* by identifying their morphological traits and examining their form using a light microscope and a field guide [16,29]. Prior to extraction, the samples were completely dried in the dark at ambient temperature (35–40 °C) [30]. For four hours, 100 g of finely powdered dried *P. australis* was submitted for extraction via 300 mL of ethyl acetate in a Soxhlet apparatus. Following the extraction, the solvent was filtered before evaporation using a rota-evaporator at 40 °C. A 0.5% yield was achieved and kept in a freezer at −80 °C for further examinations.

### 2.2. Characterization of P. australis Extract

The concentration of phenolic content in *P. australis* extract was specified spectrophotometrically using a Folin–Ciocalteau assay and the result was represented as gallic acid equivalents in milligrams per gram of the dried sample. The concentration of phlorotannin matter was determined using a DMBA (7,12−Dimethylbenzanthracene) assay and the final result was depicted as phloroglucinol equivalents in milligrams per gram of dry extract [31].

The GC-MS analysis of *P. australis* extract was performed using a Shimadzu QP-5050A GC–MS system containing a DB-1 fused silica column (60 m × 0.25 mm i.d., film thickness 0.25 μM). The column temperature was kept at 60 °C for 1 min, programmed to increase to 290 °C at 8 °C/min and was kept constant for 3 min. Other GC-MS conditions were as follows: injector temperature at 280 °C; carrier gas, helium at 1.3 mL/min; split ratio, 1:10; ionization energy, 70 eV; scan time, 1 s; mass range, 30–600 amu. Qualitative analysis of the constituents relied on a direct comparison between the retention times as well as mass spectral data and those of standard compounds, followed by computer matching with the NIST 21, NIST 107, and WILEY229 library, as well as comparing the fragmentation patterns of the mass spectra with those given out previously [32].

### 2.3. Diet Preparation

Four isoproteic (33.21% crude protein) and isolipidic (10.30%) diets were formulated to incorporate 0.0 (CTR), 100 (PA1), 200 (PA2), and 400 (PA3) mg/kg of *P. australis* extract. The ingredients (Table 1) were ground and mixed using a mixer. Lipid ingredients and *P. australis* extract were added and homogenized according to the previously mentioned quantities. The final mixtures were pelleted into 2–3 mm, air-dried at room temperature (35–40 °C) for 24 h, and preserved in plastic bags at −20 °C for future use.

### 2.4. Experimental Procedure

For this study, a total number of 300 healthy common carp weighing 14.8 ± 0.03 g were obtained from a local fish farm in Amol, Iran. Fish were examined carefully and acclimatized to indoor aquariums for two weeks and fed with CTR diet until satiation four times a day. Fish were distributed in 12 glass tanks (300 L volume) each in three replicates (25 fish/tank). Fish were fed the experimental diets for eight weeks four times a day at 3.5% body weight. The water quality was maintained in conditions that follow: water temperature 24.8 ± 0.5 °C; dissolved oxygen > 6.8 mg/L; total ammonia nitrogen (N-NH_4_) < 0.5 mg/L; pH 7.1–7.4 and a volume of 30% of the water was changed daily.

### 2.5. Growth Performance

Different growth indices, including weight gain rate (WGR), condition factor (CF), specific growth rate (SGR), and feed conversion ratio (FCR), as well as survival rate (SR), were assessed in the final stage of the experiment using the following equations:

WGR (%) = final weight − initial weight / initial weight × 100

CF = final weight / (final length)^3^ × 100

SGR = Ln final weight − Ln initial weight/feeding days × 100

FCR = dry diet feed / wet weight gain

SR (%) = final number of fish / initial number of fish × 100

### 2.6. Sample Collection

Four fish from each tank were anesthetized with 0.2 ml/L clove oil, then immediately the entire intestines were removed and kept at −20 °C in RNA-later solution (Invitrogen, Waltham, MA, USA). Fillets from the dorsal parts of the same fish were collected for proximate analysis including crude proteins, crude lipids, moisture, and ash content [33]. Blood samples were collected from the caudal veins of three fish per tank (nine fish per each group), allowed to clot at 4 °C for four hours, centrifuged at 1000 g for 10 min and finally, the separated serum samples were kept in a sterile Eppendorf tube at −20 °C before immunological assays. For the purpose of digestive enzyme assay, intestine samples from the same fish were manually removed, frozen inside liquid nitrogen, and homogenized. The intestinal homogenates were suspended in Tris-HCl (25 mM, pH 7.2). 

### 2.7. Digestive Enzyme Activities

The function of amylase was evaluated by conforming to the method described by Bernfeld [34]. Fish intestinal homogenate (50 μL) was incubated with soluble starch (1%) for 30 min. Dinitrosalicylic acid (1%) was added, and after boiling and cooling, distilled water (5 mL) was added to this mixture and the absorbance was registered at 540 nm. The lipase activity was measured according to the method described by Bülow and Mosbach [35]. The reaction buffer was composed of Tris-HCl (50 mM, pH 8.0) mixed with intestinal homogenate (20 μL) and 50 mM p-nitrophenyl butyrate (60 μL), and absorbance was recorded at 405 nm for 5 min. Trypsin and chymotrypsin activities were also measured using the commercial kit (Eastbiopharm Co. China). All intestinal enzyme activities were expressed in units/mg of fish intestine content.

### 2.8. Immune Assays

The lysozyme concentration was determined using the procedure reported by Demers and Bayne [36]. Briefly, the standard suspension of *Micrococcus lysodeikticus* (75 μg/ml) (Sigma, USA), produced in phosphate citrate buffer (0.1 M, pH 5.8) (75 μL), was added to the serum samples (25 μL) and absorbance was recorded after 4 and 9 min at 450 nm. One unit of lysozyme activity was considered as a decline in absorbance of 0.001 per min.

Serum alternative complement titer was measured according to the method described by Andani et al. [37]. Briefly, rabbit red blood cells adjusted to 2 × 10^8^ cells/ml were mixed with the fish serum samples (250 µL). After incubation for 80 min at 20 °C, NaCl solution (0.85%) was added and centrifuged (1600 g for 10 min). The amount of fish serum that yielded 50% hemolysis of red blood cells was considered as a unit of fish complement titer per ml of serum.

Serum total immunoglobulin (Ig) level was gauged according to the method described by Siwicki et al. [38]. The protein content of blood samples was determined before and after precipitation with polyethylene glycol (12%) by applying the Bradford assay, as ascribed by Kielkopf et al. [39].

### 2.9. Real-Time PCR Analysis

The intestine (30 mg) samples were processed in Gene all reagent (Gene All Biotechnology, South Korea) for RNA extraction. The concentration and integrity of the purified RNA were checked using a Bio-Rad spectrophotometer (Bio-Rad, CA, USA) and 1% agarose gel, respectively. Then, 2.5 μg RNA from purified RNA per 20 μl reaction was employed as a basis for cDNA synthesis. The cDNA reverse transcription kit (Thermo Fisher Scientific, USA) and RNase inhibitor were used to reverse the extracted RNA. The real-time PCR was performed to amplify the intestinal immune-relevant genes (*Nrf-2*, *TLR-2*, *MyD-88*, *IL-1β*, *lysozyme-C, C-3*) as well as the integrity of the relevant target genes (*Occludin, Claudin-3, Claudin-7, ZO-1* (*Zona occluden-1)*) and the reference gene (*β*-actin) (Table 2). The reaction mixture (20 μL) consisted of cDNA template (1 μl), forward and reverse primers (20 pmol), and SYBR® master mix (Takara Biotechnology Company, China) (10 μl). Amplification conditions were as follows: initial denaturation 3 min at 95 °C; cycling step 40 cycles of 20 s at 95 °C, 30 s at 60 °C; extension 20 s at 72 °C. The reaction without the cDNA template was performed as the negative control. Meanwhile, the applied primer efficiency was calculated using the following equation: E = −1 + 10^(−1/slope)^. It was discovered that the reported primer efficiencies ranged between 93 and 96%. The relative gene expression related to different target genes was calculated using the 2^−ΔΔCT^ method [40].

### 2.10. Challenge Test

When the 56-day feeding trial ended, a challenge test with *A. hydrophila* (ATCC 7966) was performed. After inoculating the bacteria in tryptic soy broth for 24 h at 30 °C, the culture was centrifuged (7000 rpm for 5 min) and the final bacterial concentration was adjusted to 1 × 10^8^ CFU/mL [41]. The remaining fish in each group (*n* = 30) were challenged intra-peritoneally with 0.1 ml of the prepared bacterial suspension. A group of fish (*n* = 30) was injected with 0.1 ml of phosphate-buffered saline (PBS) buffer and were considered as the control. Cumulative mortality in each group was recorded during 14 days, and the causative agent was examined by plucking *A. hydrophila* from the moribund fish again.

### 2.11. Statistical Analysis

Statistical analysis was conducted by means of SPSS 26.0 software. The obtained results were in the form of mean ± SE (standard error). The data differences between the groups were analyzed using one-way analysis of variance (ANOVA) and Bonferroni. The orthogonal polynomial contrast was employed to estimate the significant linear and quadratic directions of *P. australis* extract dietary levels. The survival curve was schemed using the Kaplan–Meier method and examined using the log-rank test. The data obtained from the measured genes were subjected to one-way ANOVA analysis followed by Dunnett′s multiple comparisons of group means to compare the *P. australis* extract-supplemented groups with the control group, as long as the ANOVA indicated significant differences. The difference was regarded as statistically significant at *p* < 0.05.

## 3. Results

### 3.1. Analysis of P. australis Extract 

**Figure 1:**
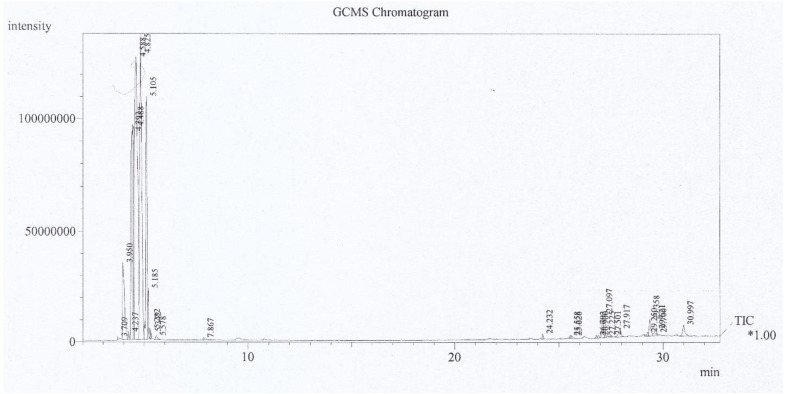
GC-MS chromatogram of *P. australis* extract. The y-axis represented the intensity count, while the x-axis represented the amount of time in minutes. Tentatively Identified Compounds (TIC) are computed using total ion areas for the TIC and the internal standard, and a relative response factor of *1.00 is assumed.

The GC-MS chromatogram of *P. australis* extract is given in Figure 1. The GC-MS analysis of the extract is also given in Table 3. This analysis shows the presence of different volatile components, including different fatty acids. The extract also contained 189.99 ± 1.1 mg/g total phenolic compounds expressed as milligram gallic acid per gram of the extract. The total phlorotannin content was about 839.43 ± 3.39 mg/g, depicted as milligram phloroglucinol per gram of this extract. 

### 3.2. Growth Performance and Carcass Composition

During this experiment, no mortality was observed in all treated groups. Fish fed *P. australis* extract had significantly higher (linear; *p* < 0.05) final weight (FW), final length (FL), condition factor (CF), and weight growth rate (WGR) when compared to the control group (Table 4). The highest values of FW, FL, and WGR were recorded in the fish group fed high levels of *P. australis* extract. Conversely, the feed conversion ratio (FCR) and specific growth rate were significantly lower in the fish group fed high doses of *P. australis* extract (quadratic; *p* < 0.05) when compared to other experimental groups. Meanwhile, no significant alterations were seen in the crude protein, lipid, moisture, and ash components (*p* > 0.05) (Table 5).

### 3.3. Digestive Enzymes

A significant enhancement was seen in lipase activity in all treatments compared to control fish (linear; *p* < 0.001) (Table 6). Moreover, the fish fed diets containing 200 and 400 mg extract/kg diet exhibited an enhancement in trypsin and chymotrypsin as compared against the other groups, but the highest trypsin activity was measured in those fed the 400 mg/kg diet (linear; *p* < 0.01). No significant alterations were seen in the amylase activity in the intestines of common carp fed different levels of *P. australis* (*p* > 0.05).

### 3.4. Innate Immune Response

The total antibody level, lysozyme activity, and serum alternative complement activity (ACH50) were meaningfully higher in common carp fed *P. australis* at 200 and 400 mg/kg diet than in the other groups (linear; *p* < 0.05) (Table 7). However, these values were higher in the fish fed 400 mg extract/kg diet than in those fed 200 mg extract/kg diet (linear; *p* < 0.05).

### 3.5. Intestinal-Integrity-Related Gene Expression

**Figure 2:**
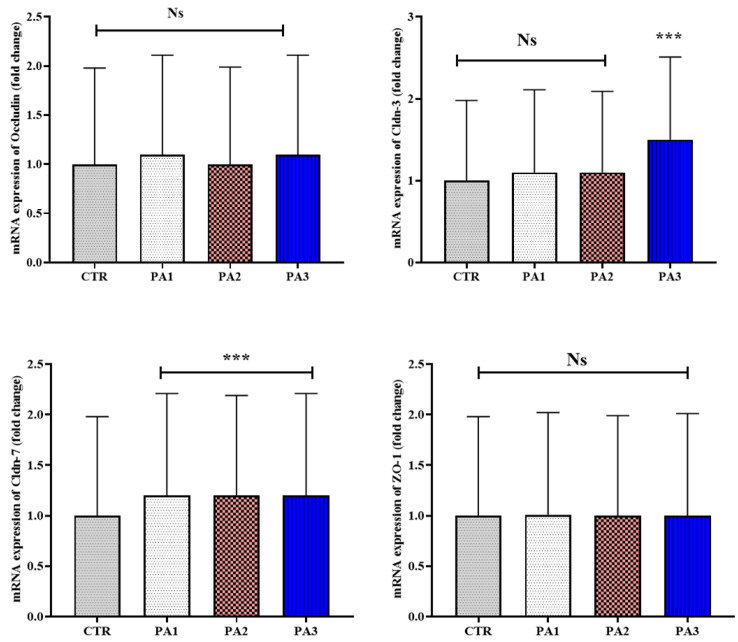
Intestinal-integrity-related gene (*Occludin*; *Claudin-3*; *Claudin-7*; *Zona occluden-1*, *Zo-1*) expressions in common carp administered with *P. australis* extract at 0.0 (CTR), 100 (PA1), 200 (PA2), and 400 (PA3) mg/diet for 56 days. Data are presented as mean ± SE (*n* = 12). Ns indicate no significant differences between groups, while *** indicates high significance at 0.001 (*p* < 0.001).

All fish groups fed diets supplemented with graded levels of *P. australis* extract demonstrated a significant upregulation of *claudin-7* gene in their intestines compared to the control fish (*p* < 0.001) (Figure 1). That being said, the transcription of *claudin-3* gene was remarkedly improved only in fish treated with 400 mg extract/kg diet (*p* < 0.001) (Figure 1). However, the inclusion of *P. australis* exhibited no significant effect on the expression of both *Occludin* and *ZO-1* genes in fish intestines (*p* > 0.05) (Figure 2).

### 3.6. Intestinal-Immunity-Related Gene Expression

**Figure 3:**
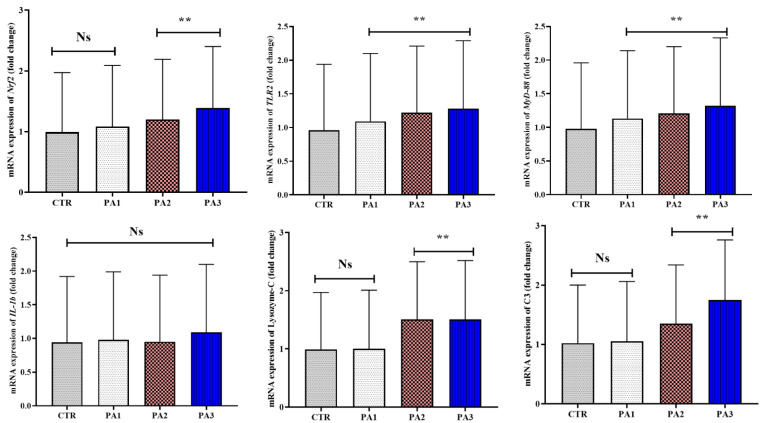
Intestinal-immune-related gene (*Nrf-2*, *TLR-2*, *MyD-88*, *IL-1β*, lysozyme-C, C-3) expressions in common carp administered with *P. australis* extract at 0.0 (CTR), 100 (PA1), 200 (PA2) and 400 (PA3) mg/diet for 56 days. Data are presented as mean ± SE. Ns indicate no significant differences between groups, while ** indicates significance at 0.005 (*p* < 0.05).

The fish fed 200 and 400 mg extract/kg diets revealed an upregulation in the expression of *Nrf-2*, *lysozyme-C*, and *C3* genes in their intestines (*p* < 0.05) (Figure 2). Additionally, expression of *Nrf-2* and C3 genes was markedly higher in fish treated with 400 mg extract/kg diet than those fed 200 mg/kg diet (*p* < 0.05). In addition, the transcription of the *TLR-2* and *MyD-88* genes was boosted in fish treated with *P. australis* regardless of the inclusion levels (*p* < 0.05) (Figure 3). The expression of *TLR-2* and *MyD-88* genes was higher in fish fed high levels of *P. australis* extract (400 and 200 mg extract/kg diets, respectively) than the other experimental groups (*p* < 0.05). Conversely, there were no significant variations in *IL-1b* expression between all experimental groups.

### 3.7. Disease Resistance

**Figure 4:**
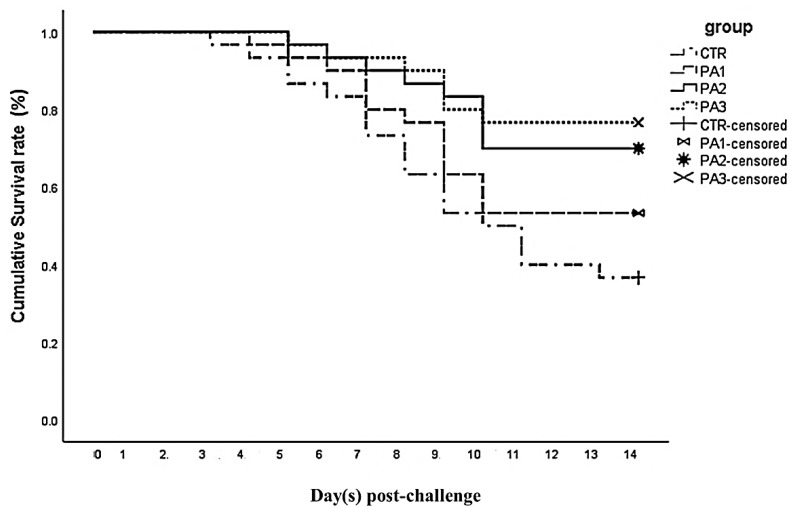
Survival rate (%) of common carp fed 100, 200 and 400 mg *P. australis* extract /kg diet and challenged with *A. hydrophila*. Differences between groups were examined using the log-rank test.

The survival rate was investigated over 14 days and the results are depicted in Figure 4. Cumulative survivals of 53.3% (*p* = 0.215), 70.0 % (*p* = 0.009), and 76.7% (*p* = 0.002) were obtained in fish fed 100, 200, and 400 mg extract/kg diets compared to 36.7% survival in the control fish (*p* = 0.134).

## 4. Discussion

Algal-derived extracts are functional additives that have vital roles in activating the immune system and inhibiting pathogenic microorganisms [17,18]. *P. australis* extracts are rich in amino acids, fatty acids, polyphenols, riboflavins, polysaccharides, minerals, and vitamins that can enhance the entire intestinal digestion process, absorption pathways, and immunity responses [22,23]. In this study, incorporating *P. australis* resulted in multiple beneficial roles in common carp, leading to high resistance to *A. hydrophila* infection. In our study, the levels of total polyphenols and total phlorotannins were similar to the findings attained by Hosny et al. [42], suggesting that ethyl acetate could be a good solvent for extracting such compounds, especially polyphenols and fatty acids, as was confirmed using different analyses. In parallel, HPLC analysis also evidenced the presence of polyphenols, including catechin in *P. australis* [43].

Our eight-week feeding trial findings show that feeding common carp fish with high levels of *P. australis* extract (200 to 400 mg/kg) improved growth performance and feed efficiency. Similarly, grey mullet (*Mugil cephalus*) administered diets supplemented with 10 mg/kg WPU (water-soluble polysaccharides extract of the green alga, *Ulva rigida*) demonstrated higher weight gain, specific growth rate, and protein efficiency ratio than the control group [44]. Such a positive effect can be in part due to the availability of different polyphenols in *P. australis* that can activate the local intestinal digestibility and mucosal immunity. *P. australis* may also set the beneficial microbiota into motion, thereby enhancing feed utilization and resulting in an increase in fish growth [27]. However, Akbary and Aminikhoei [25] have shown that polysaccharides contained in high concentrations in seaweed (*P. australis*) extract stimulate the development of beneficial bacteria, enhance gut health, and promote the growth function of the western white leg shrimp (*Litopenaeus vannamei*). In the same context, gastrointestinal enzymes play crucial roles in the digestive process, resulting in the great impact on the fish general wellbeing [45]. Our findings demonstrated that the fish group fed higher doses of *P. australis* extract had higher lipase, trypsin, and chymotrypsin activities in the intestinal digestive system. This indicates that adding 400 mg/kg. *P. australis* extract into fish diet might alter feeding transit time through the digestive tract. The reduction in transit time may have benefited digestive enzymes and may have enhanced overall digestive efficiency [46,47]. In addition, the known antioxidant capability of *P. australis* extract supplements may be linked to another explanation for increased digestive enzymatic activity in this study. It is well known that *P. australis* extract includes antioxidant components such as flavonoids, alkaloids, saponin, and steroids [48], which may be responsible for these biological benefits [49]. Therefore, an increase in the quantity of digestive enzymes such as lipase, trypsin, and chymotrypsin in the intestines of fish fed higher dietary levels of *P. australis* extract may also result in increased growth in common carp.

The health of the fish′s entire body is strongly correlated with the health status of the intestines, where the digestion and absorption of nutrients occur [50,51]. Furthermore, the intestines are one of the body gates responsible for protecting against infections induced by pathogenic invaders that can impair the innate immune system [52]. The health condition of the intestines is correlated with structural integrity, which is controlled by the tight junction proteins that can maintain the integrity of the intestines and inhibit pathogens and endotoxins from accessing the entire body through the intestines [53]. Substances such as occludin, claudins, cadherin, Zona occluden (Zos), and mucins are reported to act as physical intestinal barrier-related molecules [54]. The upregulation of claudin-3 and claudin-7 could further improve fish health status, resulting in better growth [55]. Similarly, previous studies revealed the polyphenols-mediated beneficial effects on intestinal tight junction formation and barrier function [56,57]. The studies stated that polyphenols could enhance the integration of the intestines via different mechanisms [58,59]. Dietary *P. australis* is rich in polyphenols that can increase the expression of claudin-3 and claudin-7, leading to magnified intestinal integrity. The upregulation of *Nrf-2, TLR-2, MyD-88*, lysozyme-C, and C3 in the intestines of common carp treated with *P. australis* also revealed an enhancement in the fish immune system leading to a better health condition that can cause an enhancement in fish growth performance. Furthermore, toll-like receptor 2 (*TLR-2*) as a critical ligand-binding pattern recognition receptor is involved in regulating proinflammatory cytokines (*IL-1β* and *TNF-α*) through the activation of myeloid differentiation primary response protein 88 (Myd88) [60]. In addition, nuclear erythroid 2-related factor 2 (*Nrf2*) modulates antioxidant-related enzymes that protect against lipid peroxidation and oxidative stress [61]. Here in our study, components such as polyphenols available in *P. australis* could increase *Nrf2, Myd88*, and *TNF-α* in common carp.

Infection with *A. hydrophila* severely affects fish farming and results in high mortality and economic loss [7]. However, the application of immunostimulants and functional additives, including algae extracts, limited the infection in aquaculture [12]. In this study, a significantly higher resistance was noted when treated common carp were challenged with *A. hydrophila* infection, which could be due to an increase in innate fish immunity, as the aforementioned immunological variables were higher in the treated fish than the control one. The protective role of *P. australis* against *A. hydrophila* has not yet been investigated in other fish species yet. However, Salosso *et al.* [24] stated that *P. australis* demonstrated high antibacterial activity against *A. hydrophila* in vitro.

## 5. Conclusions

In conclusion, supplemented common carp diets with *P. australis* extract at levels 200–400 mg/kg diet could promote growth, feed efficiency, digestive enzyme activities, and serum immunity; however, the maximum impacts were observed at the higher dose. Positive growth performance in fish intestines has been linked to the higher upregulation of tight junction proteins (specifically claudin-3 and claudin-7). Similarly, incorporating larger amounts of *P. australis* extract into carp fish diet significantly increased resistance to *A. hydrophila* infection and stimulated fish intestinal immune response. 

## Figures and Tables

**Table 1:** Ingredients and proximate composition of the control diet used in this study.

Ingredients	%	Proximate Analysis	
Fish meal (61.6%) Soybean meal (44.2%) Wheat flour Cotton seed meal Rice bran Corn flour Cellulose Zeolite Soy lecithin Vitamin premix ^1^ Mineral premix ^2^	30 17 8 20 4 8 2 1 4 3 3	Crude protein (%) Crude lipid (%) Dry matter (%) Ash (%) Gross energy (kcal/kg)	33.21 10.30 87.46 8.08 4167.21

^1^: Composition of mineral premix kg^−1^: calcium carbonate as carrier up to 1 kg for zinc,40 g; iron, 20 g; copper, 2.7 g; iodine, 0.34 g; manganese, 53 g; selenium, 70 mg and cobalt, 70 mg. ^2^: Composition of vitamin premix kg^−1^: vitamin B1, 700 mg; vitamin B2, 3500 mg; vitamin B6, 1000 mg; vitamin B12, 7 mg; vitamin A, 8,000,000 IU; vitamin D3, 2,000,000 IU; vitamin E, 7000 mg; vitamin K3, 1500 mg; biotin, 50 mg; folic acid, 700 mg; nicotinic, 20,000 mg; pantothenic acid, 7000 mg.

**Table 2:** Primers used for real-time PCR analysis.

Function	Target Gene	Accession Number	Annealing Temperature (°C)	Efficacy (%)	Product Size (bp)	Primer Sequences
	*Nrf-2*	JX462955	60	95	158	TTCCCGCTGGTTTACCTTAC CGTTTCTTCTGCTTGTCTTT
	*TLR-2*	HQ731681	60	95	94	GTGCTCCTGTGAGTTTGTATCT TGGTGTGTCGCACACATAATAG
	*MyD-88*	HQ380208	58	94	107	GCCCAGGAACTCACTCTAAAC GGGTCTGGTGTAATCACAGATG
Immune-related genes	*IL-1β*	KC008576.1	60	93	189	CTCTACCTTGCTTGTACCCAG AGCTGTGCTAATAAACCATCCAG
	*Lysozyme-C*	AB027305	60	96	359	GTGTCTGATGTGGCTGTGCT TTCCCCAGGTATCCCATGAT
	*C3*	AB016211	60	98	155	CAATGCCCGAGTGTCCTA TCGTTCACAGGTGTAGCC
	*Occludin*	KF975606.1	55.5	93	145	ATCGGTTCAGTACAATCAGG GACAATGAAGCCCATAACAA
	*Cldn-3*	JQ767157	56.6	94	114	GCACCAACTGTATCGAGGATG GGTTTGCCACCCAAGCCACCGGAATGA
Tight junction-related genes	*Cldn-7*	JQ767155	56	94	104	CTTCTATAACCCCTTCACACCAG ACATGCCTCCACCCATTATG
	*ZO-1*	KY290394	65	93	107	AGGAAGTTCTCCCTCGTACTC CCTCTGTTGTGGTTGAGTGTAG
Housekeeping gene	*β-actin*	M24113.1	60	93	110	TCACCACCACAGCCGAGAG CAGGGAGGAGGAGGAAGCAG

**Table 3:** GC-MS analysis of *P. australis* extract.

Retention Time (min)	Compound Name	Yield (%)
23.611	n-Eicosane	0.72
23.748	Limonen dioxide	0.92
24.142	7-oxabicyclo [4.1.0] heptane, 1-methyl-4-(2-methyloxiranyl)	1.13
24.292	Myristic acid	6.25
25.085	n-Heptadecane	0.61
25.575	Hexahydrofarnesyl acetone	0.82
25.644	Myristaldehyde	2.56
26.849	9-Hexadecenoic acid	3.69
26.961	Oleic acid	4.2
27.199	Palmitic acid	36
27.364	Arachidic acid	3.1
27.525	Ethyl palmitate	5.43
27.701	Behenic acid	2.05
27.812	Stearic acid	1.43
29.168	Phytol	0.62
29.406	9-Hexadecenoic acid	15.79
29.651	n-Octadecenoic acid	3.08
29.723	Ethyl 9-octadeccnoet	1.85
31.012	1-Hentetracontanol	9.03
32.084	n-Octadecyl isocyanate	0.72

**Table 4:** The effects of *P. australis* extract (0, 100, 200, and 400 mg extract/kg diet) dietary administration on growth performance and feed efficiency of common carp for 56 days (*n* = 3).

Parameters	Experimental Groups				*p* Value		
	CTR	PA1	PA2	PA3	Combined	Linear	Quadratic
**IW (g)**	14.75 ± 0.03	14.78 ± 0.04	14.78 ± 0.04	14.78 ± 0.03	0.314	0.339	0.289
**IL (cm)**	5.23 ± 0.02	5.23 ± 0.03	5.25 ± 0.04	5.24 ± 0.03	0.284	0.285	0.283
**FW (g)**	31.19 ± 0.08 ^c^	31.97 ± 0.59 ^c^	35.32 ± 0.75 ^b^	39.34 ± 0.92 ^a^	0.021	0.019	0.077
**FL (cm)**	12.78 ± 0.03 ^b^	12.87 ± 0.17 ^b^	13.31 ± 0.16 ^a^	13.70 ± 0.19 ^a^	0.004	0.002	0.086
**WGR (%)**	111.51 ± 2.76 ^c^	116.50 ± 3.97 ^c^	138.97 ± 2.97 ^b^	166.18 ± 2.18 ^a^	0.015	0.068	0.014
**CF**	1.49 ± 0.01 ^b^	1.50 ± 0.01 ^b^	1.50 ± 0.01 ^b^	1.53 ± 0.02 ^a^	0.034	0.033	0.071
**SGR (%/d)** ^**2**^ **FCR (g/g)** **SR (%)**	1.34 ± 0.01 ^a^ 1.86 ± 0.02 ^a^ 100	1.38 ± 0.13 ^a^ 1.81 ± 0.03 ^a^ 100	1.56 ± 0.19 ^b^ 1.74 ± 0.03 ^b^ 100	1.75 ± 0.11 ^b^ 1.66 ± 0.02 ^c^ 100	0.014	0.287	0.012
					0.002	0.084	0.002
					0.055	0.061	0.059

CTR: control group fed basal diet; PA1: the fish group fed diets supplemented with 100 mg *P. australis* extract per kg diet; PA2: the fish group fed diets supplemented with 200 mg *P. australis* extract per kg diet; PA1: the fish group fed diets supplemented with 400 mg *P. australis* extract per kg diet; IW: initial weight; IL: initial length; FW: final weight; FL: final length; WGR: weight gain rate; CF: condition factor; SGR: specific growth rate; FCR: feed conversion ratio; SR: survival rate. Data in a row superscripted by different letters are significantly different (*p* < 0.05). Data are presented as mean ± SE.

**Table 5:** The effects of *P. australis* extract (0, 100, 200, and 400 mg extract/kg diet) dietary administration on fillet composition (% wet weight basis) of common carp for 56-days (*n* = 12).

Parameters	Experimental Groups				*p* Value		
	CTR	PA1	PA2	PA3	Combined	Linear	Quadratic
Crude protein	18.28 ± 0.02	18.25 ± 0.02	18.30 ± 0.03	18.30 ± 0.03	0.587	0.594	0.601
Crude lipid	3.34 ± 0.02	3.39 ± 0.03	3.36 ± 0.02	3.34 ± 0.02	0.354	0.349	0.299
Ash	0.92 ± 0.01	0.91 ± 0.01	0.92 ± 0.01	0.92 ± 0.01	0.076	0.078	0.081
Moisture	76.20 ± 0.04	76.26 ± 0.03	76.29 ± 0.03	76.27 ± 0.03	0.094	0.096	0.091

CTR: control group fed basal diet; PA1: the fish group fed diets supplemented with 100 mg *P. australis* extract per Kg diet; PA2: the fish group fed diets supplemented with 200 mg *P. australis* extract per kg diet; PA1: the fish group fed diets supplemented with 400 mg *P. australis* extract per kg diet. Data in a row superscripted by different letters are significantly different (*p* < 0.05). Data are presented as mean ± SE.

**Table 6:** The effects of *P. australis* extract (0, 100, 200, and 400 mg extract/kg diet) dietary administration on intestinal enzyme activities (U/mg protein) of common carp for 56 days (*n* = 9).

Parameters	Experimental Groups				*p* Value		
	CTR	PA1	PA2	PA3	Combined	Linear	Quadratic
Amylase	10.26 ± 0.09	10.29 ± 0.08	10.31 ± 0.12	10.38 ± 0.14	0.241	0.257	0.231
Lipase	1.16 ± 0.12 ^c^	2.02 ± 0.09 ^b^	2.15 ± 0.28 ^a^	2.04 ± 0.10 ^b^	0.016	0.0001	0.087
Trypsin	0.223 ± 0.09 ^c^	0.224 ± 0.08 ^c^	0.246 ± 0.01 ^b^	0.270 ± 0.22 ^a^	0.030	0.011	0.068
Chymotrypsin	0.076 ± 0.21 ^b^	0.082 ± 0.17 ^b^	0.095 ± 0.12 ^a^	0.091 ± 0.15 ^a^	0.011	0.009	0.099

CTR: control group fed basal diet; PA1: the fish group fed diets supplemented with 100 mg *P. australis* extract per Kg diet; PA2: the fish group fed diets supplemented with 200 mg *P. australis* extract per kg diet; PA1, the fish group fed diets supplemented with 400 mg *P. australis* extract per kg diet. Data in a row superscripted by different letters are significantly different (*p* < 0.05). Data are presented as mean ± SE.

**Table 7:** The effects of *P. australis* extract (0, 100, 200, and 400 mg extract/kg diet) dietary administration on serum immune indices of common carp for 56 days (*n* = 9).

Parameters	Experimental Groups				*p* Value		
	CTR	PA1	PA2	PA3	Combined	Linear	Quadratic
**TIg (%)**	16.09 ± 0.19 ^c^	16.07 ± 0.13 ^c^	17.15 ±0.21 ^b^	17.43 ± 0.17 ^a^	0.005	0.036	0.058
**LYZ (μg/mL)**	28.91 ± 0.88 ^c^	30.17 ± 0.92 ^c^	39.33 ± 0.69 ^b^	44.90 ± 1.46 ^a^	0.017	0.042	0.112
**ACH50 (Unit/mL)**	115.3 ± 2.36 ^c^	122.8 ± 3.41 ^c^	130.3 ± 3.97 ^b^	144.2 ± 2.53 ^a^	0.002	0.031	0.097

CTR: control group fed basal diet; PA1: the fish group fed diets supplemented with 100 mg *P. australis* extract per kg diet; PA2: the fish group fed diets supplemented with 200 mg *P. australis* extract per kg diet; PA1: the fish group fed diets supplemented with 400 mg *P. australis* extract per kg diet; TIg: total immunoglobulin; LZY: lysozyme activity; ACH50: alternative complement pathway activity. Data in a row superscripted by different letters are significantly different (*p* < 0.05). Data are presented as mean ± SE.

## Data Availability

All data collected and analyzed during the current study are available from the corresponding author on fair request.

## References

[1] FAO (2020). The State of World Fisheries and Aquaculture.

[2] Khalafalla M.M., Zayed N.F.A., Amer A.A., Soliman A.A., Zaineldin A.I., Gewaily M.S., Hassan A.M., Van Doan H., Tapingkae W., Dawood M.A.O. (2022). Dietary *Lactobacillus acidophilus* ATCC 4356 Relieves the Impacts of Aflatoxin B_1_ Toxicity on the Growth Performance, Hepatorenal Functions, and Antioxidative Capacity of Thinlip Grey Mullet (*Liza ramada*) (Risso 1826). Probiotics Antimicrob. Proteins.

[3] Gharib A.A., Abdel-Hamid E.A., Mousa M.A., Naiel M.A. (2022). Improving water quality, growth performance, and modulating some stress physiological biomarkers in Cyprinus carpio using raw date nuclei as a zinc adsorbent agent. Appl. Water Sci..

[4] Esam F., Khalafalla M.M., Gewaily M.S., Abdo S., Hassan A.M., Dawood M.A.O. (2022). Acute ammonia exposure combined with heat stress impaired the histological features of gills and liver tissues and the expression responses of immune and antioxidative related genes in Nile tilapia. Ecotoxicol. Environ. Saf..

[5] Mugwanya M., Dawood M.A.O., Kimera F., Sewilam H. (2022). Anthropogenic temperature fluctuations and their effect on aquaculture: A comprehensive review. Aquac. Fish..

[6] Raza S.H.A., Abdelnour S.A., Alotaibi M.A., AlGabbani Q., Naiel M.A., Shokrollahi B., Noreldin A.E., Jahejo A.R., Shah M.A., Alagawany M. (2022). MicroRNAs mediated environmental stress responses and toxicity signs in teleost fish species. Aquaculture.

[7] El-Sherbeny E.M.E., Khoris E.A., Kassem S. (2022). Assessment the efficacy of some various treatment methods, in vitro and In Vivo, against *Aeromonas hydrophila* infection in fish with regard to side effects and residues. Comp. Biochem. Physiol. Part C Toxicol. Pharmacol..

[8] Mehrinakhi Z., Ahmadifar E., Sheikhzadeh N., Moghadam M.S., Dawood M.A. (2021). Extract of grape seed enhances the growth performance, humoral and mucosal immunity, and resistance of common carp (*Cyprinus carpio*) against *Aeromonas hydrophila*. Ann. Anim. Sci..

[9] Hossain A., Habibullah-Al-Mamun M., Nagano I., Masunaga S., Kitazawa D., Matsuda H. (2022). Antibiotics, antibiotic-resistant bacteria, and resistance genes in aquaculture: Risks, current concern, and future thinking. Environ. Sci. Pollut. Res..

[10] Choi W., Moniruzzaman M., Bae J., Hamidoghli A., Lee S., Choi Y.-H., Min T., Bai S.C. (2022). Evaluation of Dietary Probiotic Bacteria and Processed Yeast (GroPro-Aqua) as the Alternative of Antibiotics in Juvenile Olive Flounder *Paralichthys olivaceus*. Antibiotics.

[11] Mousavi S., Sheikhzadeh N., Tayefi-Nasrabadi H., Alizadeh-Salteh S., Khani Oushani A., Firouzamandi M., Mardani K. (2020). Administration of grape (*Vitis vinifera*) seed extract to rainbow trout (*Oncorhynchus mykiss*) modulates growth performance, some biochemical parameters, and antioxidant-relevant gene expression. Fish Physiol. Biochem..

[12] Dawood M.A.O., Koshio S., Esteban M.Á. (2018). Beneficial roles of feed additives as immunostimulants in aquaculture: A review. Rev. Aquac..

[13] Ramezanzadeh S., Abedian Kenari A., Esmaeili M. (2020). Immunohematological parameters of rainbow trout (*Oncorhynchus mykiss*) fed supplemented diet with different forms of barberry root (*Berberis vulgaris*). Comp. Clin. Pathol..

[14] Purcell-Meyerink D., Packer M.A., Wheeler T.T., Hayes M. (2021). Aquaculture Production of the Brown Seaweeds *Laminaria digitata* and *Macrocystis pyrifera*: Applications in Food and Pharmaceuticals. Molecules.

[15] Duarte C.M., Bruhn A., Krause-Jensen D. (2021). A seaweed aquaculture imperative to meet global sustainability targets. Nat. Sustain..

[16] Negm S.S., Ismael N.E., Ahmed A.I., Asely A.M.E., Naiel M.A. (2021). The efficiency of dietary *Sargassum aquifolium* on the performance, innate immune responses, antioxidant activity, and intestinal microbiota of Nile Tilapia (*Oreochromis niloticus*) raised at high stocking density. J. Appl. Phycol..

[17] Saeed M., Arain M.A., Ali Fazlani S., Marghazani I.B., Umar M., Soomro J., Bhutto Z.A., Soomro F., Noreldin A.E., Abd El-Hack M.E. (2021). A comprehensive review on the health benefits and nutritional significance of fucoidan polysaccharide derived from brown seaweeds in human, animals and aquatic organisms. Aquac. Nutr..

[18] Deepitha R.P., Xavier K.A.M., Layana P., Nayak B.B., Balange A.K. (2021). Quality improvement of pangasius fillets using aqueous seaweed (*Padina tetrastromatica*) extract. LWT.

[19] Zeilab Sendijani R., Abedian Kenari A., Smiley A.H., Esmaeili M. (2020). The effect of extract from dill *Anethum graveolens* on the growth performance, body composition, immune system, and antioxidant system of rainbow trout. N. Am. J. Aquac..

[20] Thépot V., Campbell A.H., Rimmer M.A., Jelocnik M., Johnston C., Evans B., Paul N.A. (2022). Dietary inclusion of the red seaweed *Asparagopsis taxiformis* boosts production, stimulates immune response and modulates gut microbiota in Atlantic salmon, *Salmo salar*. Aquaculture.

[21] Pradhan B., Bhuyan P.P., Patra S., Nayak R., Behera P.K., Behera C., Behera A.K., Ki J.-S., Jena M. (2022). Beneficial effects of seaweeds and seaweed-derived bioactive compounds: Current evidence and future prospective. Biocatal. Agric. Biotechnol..

[22] Yuguchi Y., Tran V.T.T., Bui L.M., Takebe S., Suzuki S., Nakajima N., Kitamura S., Thanh T.T.T. (2016). Primary structure, conformation in aqueous solution, and intestinal immunomodulating activity of fucoidan from two brown seaweed species *Sargassum crassifolium* and *Padina australis*. Carbohydr. Polym..

[23] Hassan I.H., Pham H.N.T., Nguyen T.H. (2021). Optimization of ultrasound-assisted extraction conditions for phenolics, antioxidant, and tyrosinase inhibitory activities of Vietnamese brown seaweed (*Padina australis*). J. Food Process. Preserv..

[24] Salosso Y., Aisiah S., Toruan L.N.L., Pasaribu W. (2020). Nutrient content, active compound and antibacterial activity of *Padina australis* against *Aeromonas hydropilla*. Pharm. J..

[25] Akbary P., Aminikhoei Z. (2018). Effects of *Padina australis* (Hauck) polysaccharide extract on growth, antioxidant and non-specific immune parameters of the western white leg shrimp, *Litopenaeus vannamei* (Boone). Iran. J. Aquat. Anim. Health.

[26] Shahraki N. (2016). Effect of Padina Astraulis Hauck Extract on Growth, Carcass Chemical Composition, Fatty Acids and Some of Liver Param-eters in Grey Mullet (*Mugil cephalus* Linnaeus, 1758) Larvae. Master’s Thesis.

[27] Akbary P., Shahraki N. (2020). Effect of dietary supplementation of *Padina astraulis* (Hauk) extract on biochemical response and digestive enzyme activities of grey mullet, *Mugil cephalus* (Linnaeus). Iran. J. Fish. Sci..

[28] Bita S., Akbary P., Soltanpur F. (2019). Effect of *Sargassum ilicifolium* alcoholic extract on growth, feed, body composition and digestive enzymatic activities in *Mugil cephalus*. Aquat. Physiol. Biotechnol..

[29] Win N.-N., Wai M.-K., Geraldino P.J.L., Liao L.M., Aye C.-T.P., Mar N.N., Hanyuda T., Kawai H., Tokeshi M. (2022). Taxonomy and species diversity of Padina (Dictyotales, Phaeophyceae) from the Indo-Pacific with the description of two new species. Eur. J. Phycol..

[30] Gupta S., Cox S., Abu-Ghannam N. (2011). Effect of different drying temperatures on the moisture and phytochemical constituents of edible Irish brown seaweed. LWT-Food Sci. Technol..

[31] Babaei Mahani Nejad S., Yousefzadi M., Soleimani S. (2020). Phlorotannins extracted from macroalgae as a new antioxidant source. Aquat. Physiol. Biotechnol..

[32] Cuilel I. (1994). Methodology for the Analysis of Vegetables and Drugs.

[33] AOAC (Association of Official Agricultural Chemists) (1995). Official methods of analysis of AOAC International, volume 1. Agriculture Chemicals, Contaminants, Drugs.

[34] Bernfeld P., Colowick S.P., Kaplan N.O. (1995). Amylase a and b. Methods in Enzymology.

[35] Bülow L., Mosbach K. (1987). The expression in *E. coli* of a polymeric gene coding for an esterase mimic catalyzing the hydrolysis of p-nitrophenyl esters. FEBS Lett..

[36] Demers N.E., Bayne C.J. (1997). The immediate effects of stress on hormones and plasma lysozyme in rainbow trout. Dev. Comp. Immunol..

[37] Andani H.R.R., Tukmechi A., Meshkini S., Sheikhzadeh N. (2012). Antagonistic activity of two potential probiotic bacteria from fish intestines and investigation of their effects on growth performance and immune response in rainbow trout (*Oncorhynchus mykiss*). J. Appl. Ichthyol..

[38] Siwicki A.K., Anderson D.P., Rumsey G.L. (1994). Dietary intake of immunostimulants by rainbow trout affects non-specific immunity and protection against furunculosis. Vet. Immunol. Immunopathol..

[39] Kielkopf C.L., Bauer W., Urbatsch I.L. (2020). Bradford assay for determining protein concentration. Cold Spring Harb. Protoc..

[40] Livak K.J., Schmittgen T.D. (2001). Analysis of Relative Gene Expression Data Using Real-Time Quantitative PCR and the 2^−ΔΔCT^ Method. Methods.

[41] He M., Liu G., Liu Y., Yang K., Qi X., Huang A., Liu T., Wang G., Wang E. (2020). Effects of geniposide as immunostimulant on the innate immune response and disease resistance in crucian carp. Aquaculture.

[42] Hosny M., Fawzy M., El-Borady O.M., Mahmoud A.E.D. (2021). Comparative study between *Phragmites australis* root and rhizome extracts for mediating gold nanoparticles synthesis and their medical and environmental applications. Adv. Powder Technol..

[43] Santoso J., Yoshie Y., Suzuki T. (2004). Polyphenolic compounds from seaweeds: Distribution and their antioxidative effect. Developments in Food Science.

[44] Akbary P., Aminikhoei Z. (2018). Effect of water-soluble polysaccharide extract from the green alga Ulva rigida on growth performance, antioxidant enzyme activity, and immune stimulation of grey mullet *Mugil cephalus*. J. Appl. Phycol..

[45] Ahmadifar E., Sheikhzadeh N., Roshanaei K., Dargahi N., Faggio C. (2019). Can dietary ginger (*Zingiber officinale*) alter biochemical and immunological parameters and gene expression related to growth, immunity and antioxidant system in zebrafish (*Danio rerio*)?. Aquaculture.

[46] Venkatramalingam K., Christopher J.G., Citarasu T. (2007). Zingiber officinalis an herbal appetizer in the tiger shrimp *Penaeus monodon* (Fabricius) larviculture. Aquac. Nutr..

[47] Naiel M.A., Khames M.K., Abdel-Razek N., Gharib A.A., El-Tarabily K.A. (2021). The dietary administration of miswak leaf powder promotes performance, antioxidant, immune activity, and resistance against infectious diseases on Nile tilapia (*Oreochromis niloticus*). Aquac. Rep..

[48] Junopia A., Natsir H., Dali S. (2020). Effectiveness of brown algae (Padina australis) extract as antioxidant agent. Journal of Physics: Conference Series.

[49] Esmaeili M., Abedian Kenari A., Rombenso A. (2017). Effects of fish meal replacement with meat and bone meal using garlic (*Allium sativum*) powder on growth, feeding, digestive enzymes and apparent digestibility of nutrients and fatty acids in juvenile rainbow trout (*Oncorhynchus mykiss* Walbaum, 1792). Aquac. Nutr..

[50] Dawood M.A.O. (2021). Nutritional immunity of fish intestines: Important insights for sustainable aquaculture. Rev. Aquac..

[51] Chen L., Feng L., Jiang W.-D., Jiang J., Wu P., Zhao J., Kuang S.-Y., Tang L., Tang W.-N., Zhang Y.-A. (2015). Intestinal immune function, antioxidant status and tight junction proteins mRNA expression in young grass carp (*Ctenopharyngodon idella*) fed riboflavin deficient diet. Fish Shellfish. Immunol..

[52] Zhang L., Feng L., Jiang W.-D., Liu Y., Wu P., Kuang S.-Y., Tang L., Tang W.-N., Zhang Y.-A., Zhou X.-Q. (2017). Vitamin A deficiency suppresses fish immune function with differences in different intestinal segments: The role of transcriptional factor *NF-κB* and *p38* mitogen-activated protein kinase signalling pathways. Br. J. Nutr..

[53] Liu W.-C., Zhu Y.-R., Zhao Z.-H., Jiang P., Yin F.-Q. (2021). Effects of Dietary Supplementation of Algae-Derived Polysaccharides on Morphology, Tight Junctions, Antioxidant Capacity and Immune Response of Duodenum in Broilers under Heat Stress. Animals.

[54] Lian P., Braber S., Garssen J., Wichers H.J., Folkerts G., Fink-Gremmels J., Varasteh S. (2020). Beyond Heat Stress: Intestinal Integrity Disruption and Mechanism-Based Intervention Strategies. Nutrients.

[55] Naiel M.A., Abd El-hameed S.A., Arisha A.H., Negm S.S. (2022). Gum Arabic-enriched diet modulates growth, antioxidant defenses, innate immune response, intestinal microbiota and immune related genes expression in tilapia fish. Aquaculture.

[56] Yang G., Bibi S., Du M., Suzuki T., Zhu M.-J. (2017). Regulation of the intestinal tight junction by natural polyphenols: A mechanistic perspective. Crit. Rev. Food Sci. Nutr..

[57] Mousavi S., Sheikhzadeh N., Hamidian G., Mardani K., Oushani A.K., Firouzamandi M., Esteban M.Á., Shohreh P. (2021). Changes in rainbow trout (*Oncorhynchus mykiss*) growth and mucosal immune parameters after dietary administration of grape (Vitis vinifera) seed extract. Fish Physiol. Biochem..

[58] Naiel M.A., Alagawany M., Patra A.K., El-Kholy A.I., Amer M.S., Abd El-Hack M.E. (2021). Beneficial impacts and health benefits of macroalgae phenolic molecules on fish production. Aquaculture.

[59] Naiel M.A., Negm S.S., Ghazanfar S., Shukry M., Abdelnour S.A. (2022). The risk assessment of high-fat diet in farmed fish and its mitigation approaches: A review. J. Anim. Physiol. Anim. Nutr..

[60] Frosali S., Pagliari D., Gambassi G., Landolfi R., Pandolfi F., Cianci R. (2015). How the Intricate Interaction among Toll-Like Receptors, Microbiota, and Intestinal Immunity Can Influence Gastrointestinal Pathology. J. Immunol. Res..

[61] Jia R., Cao L.-P., Du J.-L., He Q., Gu Z.-Y., Jeney G., Xu P., Yin G.-J. (2020). Effects of high-fat diet on antioxidative status, apoptosis and inflammation in liver of tilapia (*Oreochromis niloticus*) via *Nrf2*, *TLRs* and *JNK* pathways. Fish Shellfish. Immunol..

